# The Method of Activating Blood and Dredging Collaterals for Reducing Chemotherapy-Induced Peripheral Neuropathy: A Systematic Review and Meta-Analysis

**DOI:** 10.1155/2019/1029626

**Published:** 2019-06-03

**Authors:** Zhaoyi Li, Huimin Jin, Qingying Yan, Leitao Sun, Harpreet S. Wasan, Minhe Shen, Shanming Ruan

**Affiliations:** ^1^The First Clinical Medical College of Zhejiang Chinese Medical University, Hangzhou, 310053, Zhejiang, China; ^2^Department of Cancer Medicine, Hammersmith Hospital, Imperial College Healthcare NHS Trust, London, UK; ^3^Department of Medical Oncology, The First Affiliated Hospital of Zhejiang Chinese Medical University, Hangzhou, 310006, Zhejiang, China

## Abstract

**Background:**

Chemotherapy-induced peripheral neuropathy (CIPN) remains as a big unsolved challenge for cancer patients and oncologists. However, there is no effective treatment to prevent and cure it. This systematic review and meta-analysis chiefly aimed to assess the effectiveness and safety on the method of activating blood and dredging collaterals in traditional Chinese medicine (TCM) for reducing CIPN.

**Methods:**

Two authors comprehensively searched all the randomized controlled trials (RCTs) via PubMed, Cochrane, China National Knowledge Infrastructure (CNKI), and Wanfang Database of China Science Periodical Database (CSPD). The Review Manager (RevMan) 5.0 was used to conduct the meta-analysis.

**Results:**

20 trials including 1481 participants were analyzed. 15 trials tested the incidence of all-grade CIPN which was significantly lower in intervention arm and 16 trails presented that the result of high-grade CIPN was the same. The total effective rate of the use of Chinese herbs was 77.19% versus 45.79% in the comparator group. Besides, the use of Chinese herbs statistically promoted the sensory nerve conduction velocity (SNCV) and the motor nerve conduction velocity (MNCV). Besides, the quality of life (QoL) in the intervention group was better than the comparator one. Herbs-related adverse events were skin allergy, skin chap, and scald, which could be managed well.

**Conclusions:**

The work involving studies of the effectiveness and safety on TCM for reducing CIPN proves to be encouraging. Herbs with the function of activating blood and dredging collaterals were found to potentially promote the curative effects as well as making improvements of SNCV and MNCV. However, in the future, more double-blind, multicenter, large-scale RCTs and more comprehensive researches are still required.

## 1. Introduction

Chemotherapy-induced peripheral neuropathy (CIPN) is an inevitable dose-limiting side effect and approximately 30–40% of patients treated with neurotoxic chemotherapy agents, such as platinum, vinca alkaloids, and taxanes, will suffer from it [1]. Pain CIPN can be extremely disabling, with a marked impact on quality of life, functional ability, and risks of noncompliance with cancer treatment. Those side effects can result in a dose reduction of chemotherapy regime, even terminating the course of treatment, thus limiting therapeutic efficacy. Shi Q et al. [2] found that high-grade CIPN (grades 3-4) was more likely to occur in 3-month treatment vs. 6-month treatment of CapeOX or FOLFOX.(3% vs. 9% for CapeOX; 3% vs. 16% for FOLFOX.) Besides, CIPN apparently increases the financial burden on patients as well as the annual costs of healthcare [3].

Research reports that paclitaxel-induced mechanical allodynia is due to the transcriptional increase in matrix metalloproteinases (MMP) 2 and 9 and decrease of metallopeptidase inhibitor 1 (TIMP1) [4]. And the route of drug administration is considered as an important factor in the development of CIPN [5, 6]. A study indicates that older age, cardiovascular drugs, and preexisting nerve compression syndromes are likely to increase neuropathy risk [7]. Conventional therapy options for CIPN include antidepressants, anticonvulsants, neuromodulation, and physical therapy [8]. A recent RCT proved that sensorimotor training and whole-body vibration training were promising to reduce symptoms [9]. Clinical reports show dorsal root ganglion (DRG) stimulation may be useful for alleviating the neuropathic pain [8]. There are 15 CIPN-directed clinical trials sponsored by the National Cancer Institute, and it is concluded that alpha-lipoic acid, intravenous calcium/magnesium, vitamin E, or glutathione acts as the prevention, and nortriptyline, gabapentin, lamotrigine, amifostine, or duloxetine acts as symptomatic treatment [10]. Although CIPN can cause dose reduction or even chemotherapy termination in cancer patients, there is still no FDA-approved validated treatment for preventing or reversing the condition of CIPN.

In recent years, Chinese herbal medicine (CHM) has been common and eligible in cancer-related symptom management. Meta-analysis showed that the combination of CHM and conventional treatment can significantly reduce the neuropathy pain [11]. Many TCM oncologists insist that one of the pathogeneses of CIPN is believed to be qi stagnation and blood stasis in collaterals, so promoting blood circulation and dredging collaterals to remove meridian obstruction contribute to the prevention and relief of CIPN. A study proved that specific Chinese herbs and their components of activating xue (blood), such as* Angelica*, tetramethylpyrazine, astragaloside, and safflower, can function as antithrombolysis and improve microcirculation in nervous system [12]. Danshen and its active constituents, tanshinone IIA (TIIA) and cryptotanshinone (CRY), were investigated to be effective in reverting chemotherapy-induced neuropathic pain [13]. Safflower extract and aceglutamide injection is investigated to be efficient in reducing the nerve injury and promoting recovery of peripheral innervations in animal test [14]. There has been some systematic review or meta-analysis about the therapeutic effect of herbal medicine such as Guilong Tongluo decoction, Yiqi Huoxue decoction, Radix Astragali-Based Chinese Herbal Medicine, or Goshajinkigan CIPN [15–17]. However, no systematic review to date has reported the method of activating blood and dredging collaterals in reducing chemotherapy-induced peripheral neuropathy. In this meta-analysis, we aim to investigate the effectiveness and safety of this traditional Chinese medicine treatment when used in prevention and treatment of CIPN. We want to discover whether using Chinese herbs will be better than other alternatives, such as placebo, no intervention, or some western medicines.

## 2. Materials and Methods

### 2.1. Database and Search Strategies

We comprehensively searched the following electronic databases using keywords of “Chemotherapy-induced peripheral neuropathy” or “CIPN”, “activating blood and dredging collaterals” or “dispersing blood stasis” or “Chinese medicine ABDC therapy” or “Danggui” or “rhizome of chuanxiong” or “safflower” or “Guizhi” or “Astragali radix” or “Chi Shao” or “Salvia” or “NiuXi” or “Maidenhair Tree” or “Ginkgophyta” etc., “Randomized Controlled Trials as Topic” or “controlled clinical trial∗” or “randomized∗” or “placebo” or “clinical trial*∗*” or “controlled trial*∗*” etc. without language or publication date limitations: PubMed, Cochrane, China National Knowledge Infrastructure (CNKI), and Wanfang Database of China Science Periodical Database (CSPD). Applying to each database, we modified the search strategies, respectively. All of those searches were completed before December 2018.

This systematic review has been registered in PROSPERO, and the registration ID is CRD42018116749.

### 2.2. Inclusion Criteria

We included all high quality randomized controlled trials (RCTs) investigating the effects of herbal medicine which had the function of activating blood and dredging collaterals for preventing and treating CIPN in cancer patients without restriction time or language. Among those trails, the number of patients in each arm was more than 15 and the Jadad score was 4-7 points.

#### 2.2.1. Participants Types

The participants were included to meet the following criteria:

(1) Age of 18 years or older

(2) Patients who were diagnosed with cancer and received chemotherapy regardless of type of cancer, sex, race and location

(3) CIPN diagnosed by clinical assessment or additional investigation such as nerve conduction velocity

#### 2.2.2. Types of Interventions

All types of herbal medicines which could activate blood and dredge collaterals were included. There were no limitations on the composition of prescription, the origin, the mode of delivery (e.g., oral, intravenous, or per cutaneous), dosage, and duration of treatment.

Control intervention would include no TCM treatment; or placebo; or conventional therapeutic agents, such as vitamin E or Ca/Mg infusions. Trials of herbal medicine meeting the standard plus conventional medicine versus the same conventional medicine alone were also included.

### 2.3. Outcome Measures

#### 2.3.1. Main Outcomes

(1) Clinical effectiveness was assessed by objective methods such as World Health Organization (WHO) grade [38], National Cancer Institute common terminology criteria for adverse events (NCI-CTCAE) CIPN grade [39], or Levi's grade [40].

(2) Complete remission (CR) meant the grade of CIPN reduced to 0 grade and all symptoms disappeared. Partial remission (PR) meant the grade of CIPN reduced more than 1 grade and the symptoms abated. The effective rate was the sum of CR and PR. Nonperceptible (NP) meant symptoms had not abated after therapy, and the grade of CIPN did not reduce.

(3) Incidence rate of CIPN was assessed by the above methods.

(4) Nerve Conduction Velocity (sensory nerve conduction velocity (SNCV) and motor sensory nerve conduction velocity (MNCV)) was evaluated after 1 week of TCM treatment or more.

#### 2.3.2. Additional Outcomes

(1) High frequency herbs

(2) The characteristics of the formulas

(3) Quality of life assessed with Karnofsky (KPS) scale or Eastern Cooperative Oncology Group (ECOG) scale

(4) Extracted incidence rates of adverse events relative to chemotherapy or herbal medicine

### 2.4. Exclusion Criteria

We excluded those trials that used the herbal medicine with other functions; whose number of patients in any arm was less than 15; that were assessed to be low quality with the Jadad score being 1-3; that had not used the same baseline therapy; or that employed the methods of acupuncture or moxibustion.

### 2.5. Risk of Bias Analysis

Two authors independently assessed the risk of bias which was described in the Cochrane handbook for systematic reviews of interventions [18]. We classified the potential bias as high, low, or unclear. The following items were assessed:

(1) Random sequence generation

(2) Allocation concealment

(3) Blinding of participants and personnel

(4) Blinding of outcome assessment

(5) Incomplete outcome data

(6) Selective outcome reporting

(7) Other bias

### 2.6. Research Selection and Statistic Collection

Two review authors screened articles based on titles and abstracts firstly after eliminating the duplicate publications. Then the full-text versions of the papers that met the inclusion criteria were retained and data on patient characteristics, treatment details, and clinical outcomes were extracted independently. Differences in opinion would be resolved by a third reviewer. Reference lists of the included studies were checked by hand.

### 2.7. Quality Assessment

Improved Jadad scale was applied to assess the quality of RCTs, and the items included randomization, blinding of participants, personnel, outcome assessors, incomplete outcome data, and other threats to validity [18]. 4–7 points represent high quality, while 1–3 points represent low quality.

### 2.8. Statistical Analysis and Data Synthesis

RevMan 5.3 software provided by Cochrane Collaboration was performed for the data analysis. We used mean difference (MD) analysis with 95% confidence intervals (CI) for continuous outcomes and risk ratios (RR) or odds ratio (OR) with 95% CI for binary outcomes. In case that different measurement scales were used, standardized mean difference (SMD) analysis with 95% CI was conducted. We tested the heterogeneity through* I*^*2*^, and it would present as significant when* I*^*2 *^was over 50% or *P* < 0.05. We would perform random effect model if there was significant heterogeneity, while fixed effect model would be used when the heterogeneity was moderate [18]. If the included studies were ≥10, funnel plots would be employed to assess reporting bias. Subgroup analyses were done based on the types of the interventions and comparator.

## 3. Results

### 3.1. Description of Studies

**Figure 1:**
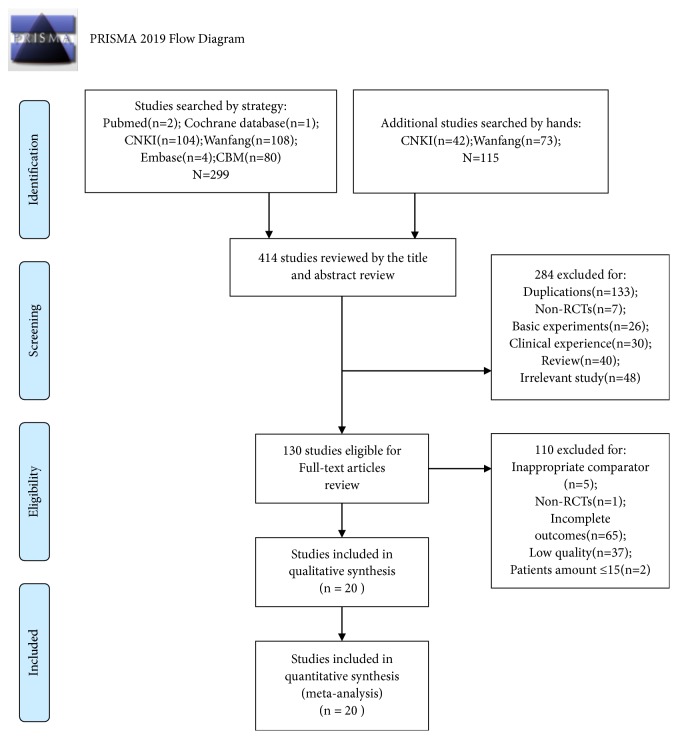
Flow diagram of the included studies.

The flow diagram was depicted as in Figure 1. We primarily identified 330 studies searched by strategy and hand from the above 4 electronic databases (Figure 1). After reviewing by the titles and abstracts, we excluded 220 studies, including 97 duplicate articles, 7 non-RCTs, 26 basic experiments, 30 clinical experiences, 39 reviews, and 21 irrelevant studies. We retrieved the full texts of 110 articles for further evaluations, of which 90 studies were excluded for the reasons of inappropriate comparator, non-RCTs, incomplete outcomes, low quality, and patients number ≤15. In the end, a total of 20 trials were included for this review [19–37, 41].

### 3.2. Characteristics of Included Studies

Table 1 shows the characteristics of included studies. In summary, a total of 1481 patients were included in 20 trials with 30 patients being dropped from the study. The mean size of the participants was 74.05 people, ranging from 36 to 128 per trial. The baseline characteristics in the included trials were comparable between the intervention groups and the comparator groups. As for the types of cancer, ten trials referred to the colorectal cancer, seven studied gastric cancer, nine tested with various types of cancer, and one studied ovarian cancer. The chemotherapy regimens in participants included oxaliplatin based chemotherapy (*n*= 9 trials), FOLFOX 4 (*n*= 4 trials), XELOX (*n*= 1 trial), cisplatin based chemotherapy (*n*= 1 trial), mFOLFOX6 (*n*= 1 trial), TP (paclitaxel and cisplatin) (*n*= 2 trials), and no specific common regimen (*n*= 2 trials).

Regarding the regimens of comparators, Cobamamide was used in one trial, Mecobalamin was used in three, a placebo was used in three, Tropisetron and dexamethasone were used in one, and hand and foot baths of warm water were used in two. The other 10 trials compared interventions with no additional treatment. The range of the treatment duration was from 7 days to 32 weeks.

Additionally, it provides information of the outcomes indexes. For the assessment of the incidence rate or the clinical improvement, 8 trials used Levi's grade, 7 used NCI-CTC grade, 4 used WHO criteria, 2 used the researcher's own criteria, and 1 used the Nimodipine Trichotomy. Besides, 8 studies reported the NCV (nerve conduction velocity) involving the MNCV (motor nerve conduction velocity) and the SNCV (sensory nerve conduction velocity).Five trials tested the QoL and 9 studies reported the adverse events.

### 3.3. Intervention Comparisons

As shown in Table 1, 10 trials compared herbs with no intervention or with placebo. Five trials tested herbs against western medications, such as Cobamamide, Thymopentin, Mecobalamin, Glutathione, Methycobal, calcium gluconate, and magnesium sulfate. Furthermore, another 5 trials tested herbs in combined regimens compared to the same western medications. In view of the administration method, three forms of dosage were employed, respectively: oral dosage form including decoction or granules (*n*=7 trials), topical administration involving hand and foot baths or fumigation or compress or gel (*n*=10 trials), and intravenous infusion (*n*=3 trials).

Table 2 demonstrates the characteristics of the 17 different formulas researched in the total 20 trials. The most frequent prescription was modified Huangqi Guizhi Wuwu Decoction (*n*=5 trials). Some prescriptions constituted by the researchers themselves were combined or transformed from it (*n*=6 trials). Others are made up of other herbs with the function of activating blood and dredging collaterals (*n*=6 trials). The high frequency Chinese herbs are shown in Table 3. The top five herbs are Guizhi (*n*=14,10.53%), Huangqi (*n*=9,6.77%), Baishao (*n*=9,6.77%), Danggui (*n*=7,5.26%), and Chuanxiong (*n*=7, 5.26%).

### 3.4. Risk of Bias Analysis Outcomes

All the results are shown in Figures 7(a) and 7(b).

#### 3.4.1. Random Sequence Generation

Eight studies were judged to be at low risk of bias for using a computer random number generator or random number table method, ten were judged to be at unclear risk of bias for not mentioning random sequence generation, and the remaining two studies were judged to be at high risk of bias for using the wrong way.

#### 3.4.2. Allocation Concealment

Eight studies were judged to be at low risk of bias for reporting allocation concealment or the allocation method having no influence on the results. Nine studies did not mention allocation concealment being judged to be at unclear risk of bias.

#### 3.4.3. Blinding of Participants and Personnel

Five trials set up placebo arm and reported blinding of patients and study personnel being judged to be at low risk of bias. Thirteen studies did not blind the study participants or personnel to be regarded as high risk of bias. Two studies were judged to be at unclear risk of bias for not mentioning it.

#### 3.4.4. Blinding of Outcome Assessors

Five studies were judged to be at low risk of bias for setting up placebo arm or blinding the data collectors or being analyzed to have little possibility to break the blinding. While the remaining fifteen studies did not mention the outcome assessors blinding, they were judged to be at unclear risk of bias.

#### 3.4.5. Incomplete Outcome Data

Patients in sixteen trials were reported to complete the whole course of treatment. Another four studies reported the reasons of the drop-out participants which were assessed to have no clinical effect on the outcome data. As a result, all the 20 studies were judged to be at low risk of bias.

#### 3.4.6. Selective Reporting

All trials were not registered anywhere and provided no information of the selective report, to be judged to be at unclear risk of bias.

#### 3.4.7. Other Bias

All the studies were judged to be at unclear risk of bias for being tested to be free of other apparent bias.

### 3.5. Therapeutic Effects

#### 3.5.1. Incidence of All-Grade CIPN (Figure 2(a))

A total of fifteen trials tested the incidence of all-grade (grades 1-4) CIPN. Among those trials, based on the types of the interventions and comparators, we did subgroup analyses. Eight trials compared herbs based intervention to no intervention or placebo after we excluded one [32] in which there was no difference in the event between intervention and comparator. Chinese herbs intervention might have promising beneficial effects in preventing or reducing CIPN occurrence (*n *= 617 patients; OR, 0.22, 95% CI, 0.14 to 0.34,* P *< 0.00001). Three trials compared Chinese herbs to western medications, including Tropisetron, dxm, and Mecobalamin. It was discovered that herbs showed beneficial influences on preventing or reducing CIPN occurrence (*n* = 142 patients; OR, 0.22, 95% CI, 0.09 to 0.54,* P *= 0.0008). In addition, four trials compared Chinese herbs plus Methycobal Injection, calcium gluconate, magnesium sulfate for injection, Glutathione, AD pro injection, and Thymopentin for subcutaneous injection with the same western medications. It was demonstrated that herbs in combined remedies significantly prevented or reduced CIPN occurrence (*n* = 334 patients; OR, 0.36, 95% CI, 0.22 to 0.59,* P *< 0.0001).

#### 3.5.2. Incidence of High-Grade CIPN (Figure 2(b))

Sixteen trials reported the incidence of high-grade (grades 3-4) CIPN. Nine trials compared herbs to no intervention or placebo. It was assessed that Chinese herbs had promising potential in preventing or reducing CIPN occurrence (*n *= 673 patients; OR, 0.34, 95% CI, 0.20 to 0.61,* P *=0.0002). Three trials compared Chinese herbs to western medications, including Tropisetron, dxm, and Mecobalamin. Four trials compared Chinese herbs plus Methycobal Injection, calcium gluconate, magnesium sulfate for injection, Glutathione, AD pro injection, and Thymopentin for subcutaneous injection with the same western medications. Although there was no significant difference between groups in the above seven trials, in the total sixteen trials, it was in favour of the intervention (*n *= 1149 patients; OR, 0.35, 95% CI, 0.22 to 0.57,* P *< 0.0001).

#### 3.5.3. Curative Effects of the Method of Activating Blood and Dredging Collaterals for Reducing CIPN (Figure 3)

To sum up, six trials including 418 participants reported curative effects of the method of activating blood and dredging collaterals for reducing CIPN. The total effective rate of the use of Chinese herbs was 77.19% versus 45.79% in the comparator group. Three studies compared curative effects of Chinese herbs to no additional treatment or placebo. Chinese herbs were proved to be more efficient in relieving CIPN (*n *= 233 patients; OR, 4.57, 95% CI, 2.48 to 8.40,* P *< 0.00001). Two trials making comparison of Chinese herbs and western medications such as Cobamamide and Mecobalamin and herbs were suggested to be effective in CIPN relief (*n *= 125 patients; OR, 4.91, 95% CI, 1.10 to 21.81,* P *=0.04). One trial compared Chinese herbs plus Mecobalamin, AD pro injection, and Thymopentin, and applying herbs was also more effective in relieving CIPN (*n *= 60 patients; OR, 4.13, 95% CI, 1.39 to 12.27,* P *=0.01).

#### 3.5.4. Sensory Nerve Conduction Velocity (SNCV)

**Figure 4:**
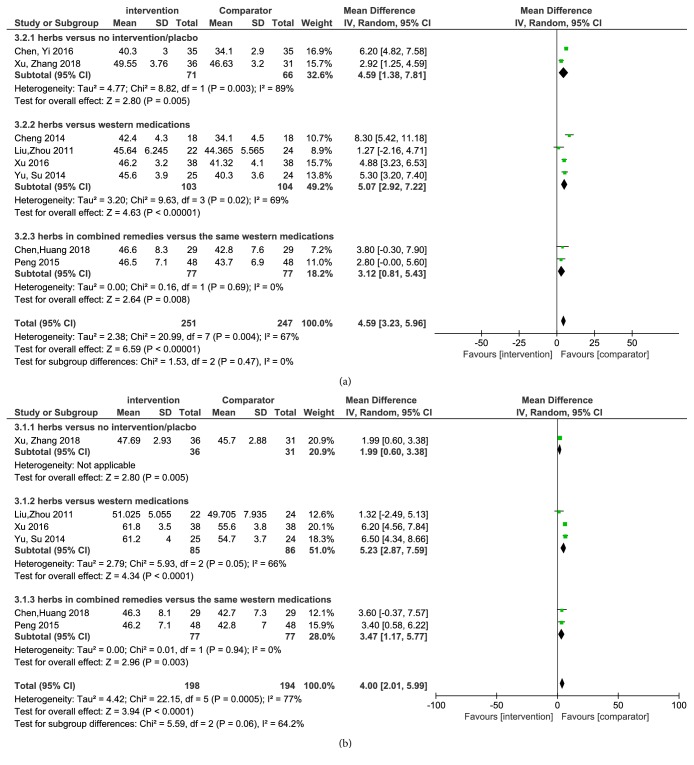
(a) Forest plot of comparison: sensory nerve conduction velocity of the fibula nerve. (b) Forest plot of comparison: sensory nerve conduction velocity of the median nerve.

As shown in Figure 4(a), eight trials analyzed the sensory nerve conduction velocity of the fibula nerve. It was attested that the Chinese herbs had beneficial influences on improving the SNCV of the fibula nerve (MD 4.59 m/s, 95% CI 3.23 to 5.96,* P*< 0.0001). Besides, Figure 4(b) indicated that six trials assessed the sensory nerve conduction velocity of the median nerve. It was discovered that the use of Chinese herbs statistically promoted the SNCV of the median nerve (MD 4.00 m/s, 95% CI 2.01 to 5.99,* P*< 0.0001).

#### 3.5.5. Motor Nerve Conduction Velocity

**Figure 5:**
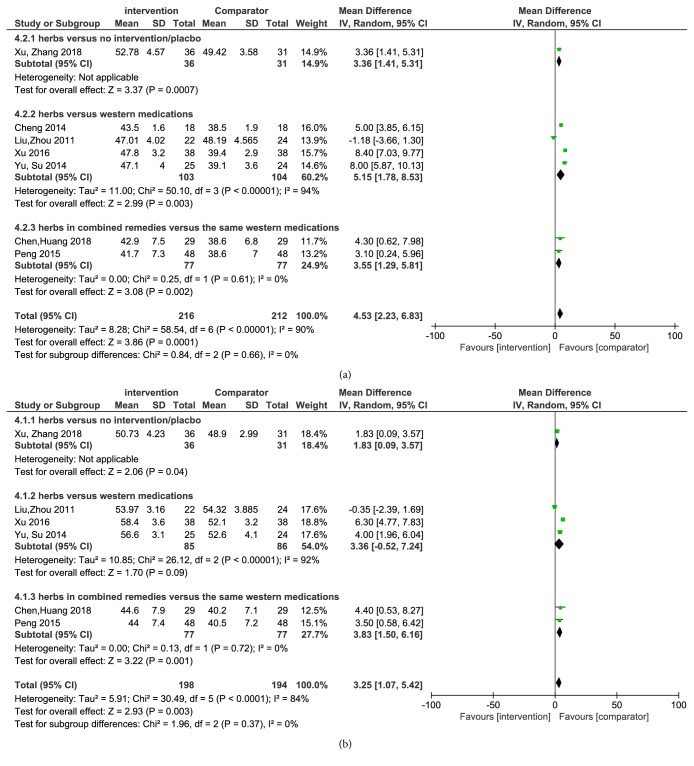
(a) Forest plot of comparison: motor nerve conduction velocity of the fibula nerve. (b) Forest plot of comparison: motor nerve conduction velocity of the median nerve.

Figure 5(a) showed seven trials tested the motor nerve conduction velocity of the fibula nerve, which proved that the Chinese herbs were more effective in improving the MNCV of the fibula nerve (MD 4.53 m/s, 95% CI 2.23 to 6.83,* P*= 0.0001). In addition, Figure 5(b) revealed that six trials detected the MNCV of the median nerve, where Chinese herbs were proved to be valid in enhancing the MNCV of the median nerve (MD 3.25 m/s, 95% CI 1.07 to 5.42,* P*< 0.0001).

### 3.6. Quality of Life (QoL) and Adverse Events

Five trials reported QoL (KPS score > 60 or ECOG score ≤ 2). Two trials [19, 26] reported the comparison of the percentage of patients with QoL improvement. Three trials [20, 25, 29] indicated differences of KPS scores or levels between two groups. One trial [32] investigated the ECOG PS based on the EORTC QLQ-C30. Thus, QoL could not be combined and analyzed in the meta-analysis. On the whole, the quality of life in the intervention group was better than the comparator one.

Among the total 20 studies, three trials [21, 24, 27] reported the herbs-related adverse events, such as skin allergy, skin chap, and scald, while three trials [20, 24, 25] showed the chemotherapy-induced adverse events, including myelosuppression, hematological toxicity, and gastrointestinal side effect.

### 3.7. Publication Bias

**Figure 6:**
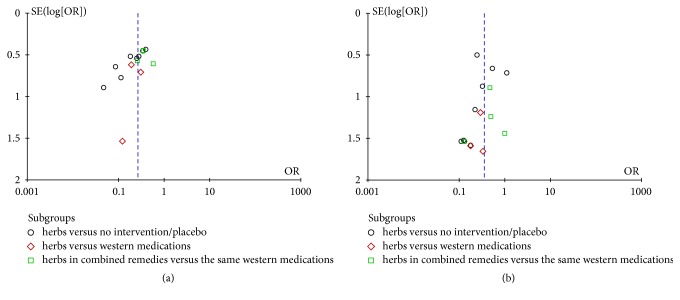
Funnel plot analysis of risk of bias. (a) Funnel plot analysis of incidence of all-grade chemotherapy-induced peripheral neuropathy (CIPN). (b) Funnel plot analysis of incidence high-grade CIPN (grades 3-4).

**Figure 7:**
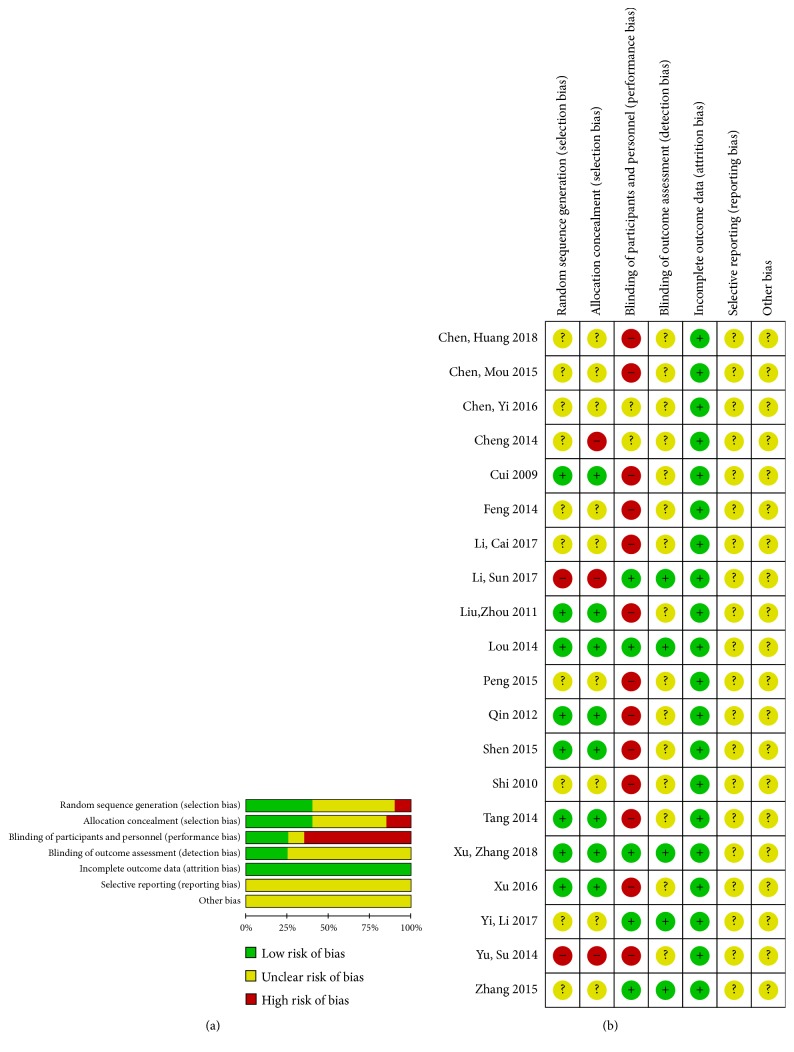
(a) Risk of bias graph. (b) Risk of bias summary: review of authors' assessment about each risk of bias item for each included study. “+”: low risk of bias; “?”: unclear risk of bias; “−”: high risk of bias.

In Figures 6(a) and 6(b), the funnel plots of the incidence comparison of all-grade CIPN and high-grade CIPN demonstrated near-symmetry. Therefore, we found no significant publication bias.

## 4. Discussions

### 4.1. Advantages

We have attempted to analyze four main outcomes to evaluate the effectiveness on CIPN of herbal medicines alone or combined with western therapies in comparison with different comparators. The words of “activating blood” mean removing blood stasis, antiplatelet aggregation, and ameliorating the blood circulation of body, etc.; “dredging collaterals” refers to improving the microcirculation in nervous system, protecting the neurons, anti-ischemic, and neurotransmitter modulatory effects, etc.

Chinese herbs selected and combined based on the method of activating blood and dredging collaterals were suggested to have a preventive and therapeutic role in reducing CIPN, not only for all-grade CIPN, but also for high-grade CIPN, as well as promoting curative effectiveness. Numerous prior works have focused on verifying it. Chen D et al.'s review [42] reported that Niuche Shenqi Wan were found to display potential therapeutic effects for preventing the genesis and development of CIPN via restoring the slowed blood flow, inhibiting oxidative stress and activating the C fiber. Liu et al.'s RCT [43] indicated that Guilong Tongluo decoction could delay the onset time of grades 1–4 neurotoxicity (9.4 vs. 6.5 weeks, P < 0.05). Interestingly, we discovered that, among the high frequency herbs, Guizhi, Huangqi, and Baishao were the main compounds of the formula Wangqi Guizhi Wuwu decoction. Huangqi Guizhi Wuwu decoction is an herbal formula recorded in “Synopsis of the Golden Chamber” for improving limb numbness and pain. And its extract AC591 was clarified to prevent oxaliplatin-induced neuropathy, such as cold hyperalgesia and mechanical allodynia as well as morphological damage of dorsal root ganglion, and might be a promising adjuvant to alleviate sensory symptoms in clinical practice [44]. Di Cesare Mannelli et al.'s animal experiment [45] drew a conclusion that 50% hydroalcoholic extract of Astragali Radix could obstruct the beginning of the proallodynia effect completely and relieve CIPN in oxaliplatin-treated rats.

As we all know, treatment according to syndrome differentiation is one of the major features in traditional Chinese medicine, which requires the heterogeneous prescriptions fitting for different patients. As discussed above, the formulation, administration, dosage, and duration of treatment were all various. It provokes the thinking on the relationship of syndrome differentiation treatment in TCM and personalized therapy in western medication for tumor.

What is more, the reasons why both SNCV and MNCV could be enhanced after using the Chinese herbal medicines might be that herbs functioned as antithrombolysis, improved microcirculation in nervous system, reduced the nerve injury, promoted recovery of peripheral innervations, and so on. Oztürk G et al.'s research [46] demonstrated the neuroprotective effect of Ginkgo extract EGb761 with faster NCVs, which was probably due to its prevention on pathological changes of decrease in somatic and nuclear size, nucleolar segregation, and multinucleolation.

Furthermore, the adverse event and KPS reports had important implications for patient management under the various use of the Chinese herbs to ensure security.

### 4.2. Disadvantages

However, this review has its limitations, which made it difficult for us to make a definite conclusion. The literature included was not comprehensive enough, excluding conference papers, dissertations, unpublished papers, and papers with Jadad less than 3 points. Another disadvantage is that RCTs included are small-scale, short-term trials. Additional subgroup analysis exploring the influence of age, chemotherapy regimen, and treatment cycle could not be implemented due to insufficient data. What was unsatisfactory was that the overall methodological quality of the studies was relatively low except for a few studies, such as Lou et al.'s research and Li, Sun et al.'s research. Surprisingly, over half of the included trials did not provide adequate clarifications for random sequences, allocation concealment, and double-blinded set.

These limitations prevented us from arriving at an accurate conclusion.

## 5. Conclusions

In general, this systematic review and meta-analysis results will hopefully serve as useful feedback information for preventing and reliving chemotherapy-induced peripheral neuropathy. Herbs with the function of activating blood and dredging collaterals were found to potentially promote the curative effects as well as making improvements of SNCV and MNCV.

But the evidence is not sufficient to draw a definite conclusion for the small participant sizes, the low methodological quality, the uncomprehensive subgroup analysis, and so on. In the future, more double-blind, multicenter, large-scale RCTs and more overall researches are still required before final goal of achieving effective improvement of CIPN by using traditional Chinese medicine can be completed.

## Figures and Tables

**Figure 2:**
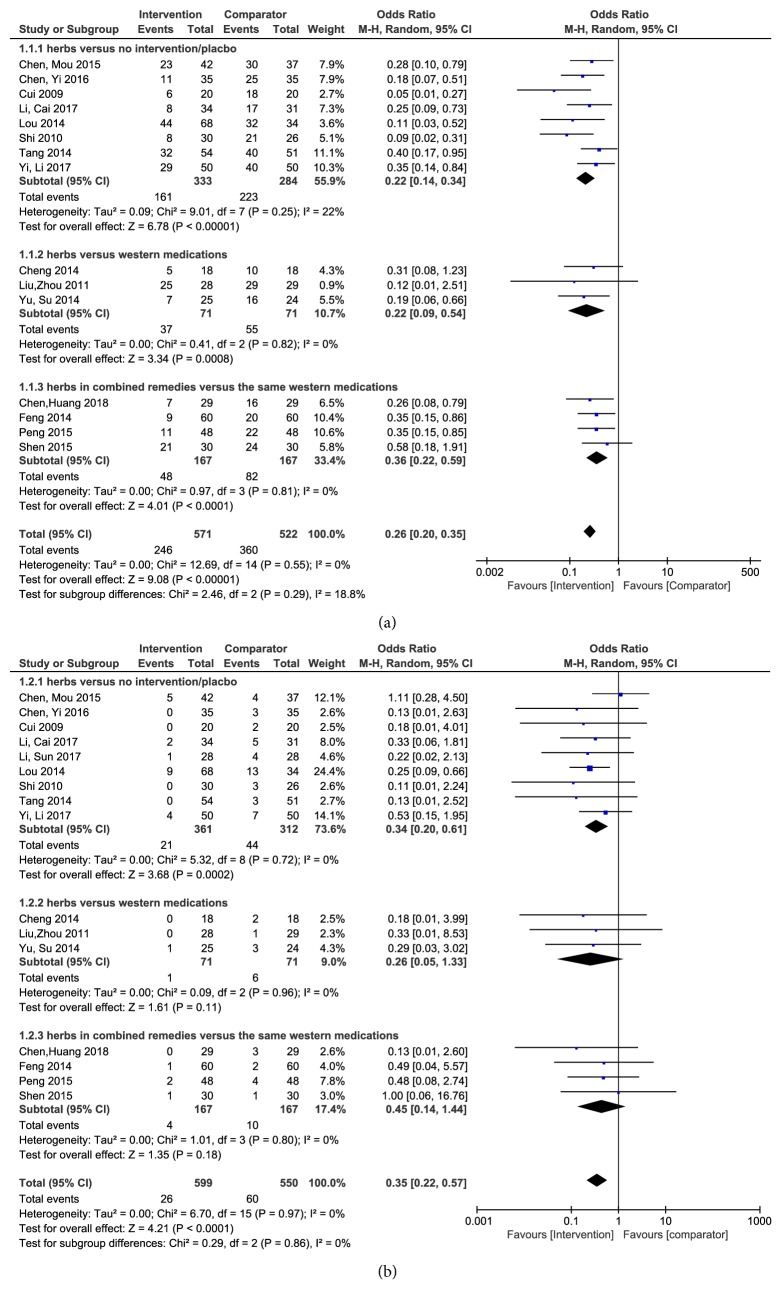
(a) Forest plot of comparison: incidence of all-grade chemotherapy-induced peripheral neuropathy. (b) Forest plot of comparison: incidence of high-grade chemotherapy-induced peripheral neuropathy.

**Figure 3:**
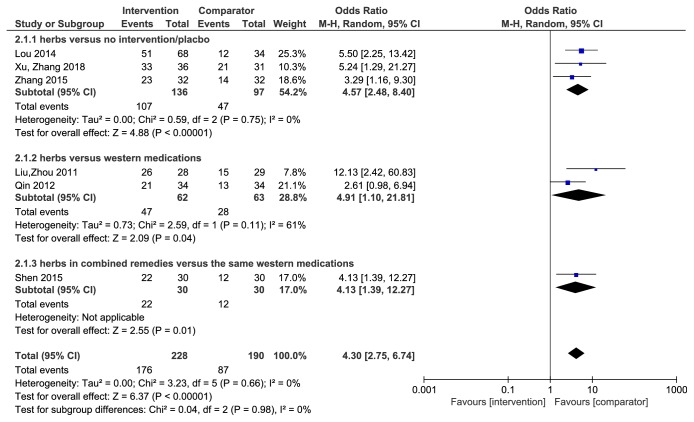
Forest plot of comparison: curative effects of the method of activating blood and dredging collaterals for reducing CIPN.

**Table 1:** Characteristics of the included studies of the systematic review.

First author	Year	Sample size (drop-out)	Mean age (year) (median/range)	Cancer Types	Common treatment (regimen)	Interventions (regimen, participants)	Comparators (regimen, participants)	Main Outcomes
*Herbal medicine for hand and foot baths or fumigation or compress or gel*								
Chen, Mou [18]	2015	79 (0)	50.12±*10.21*	Gastric and colorectal cancer	FOLFOX 4	Hand and foot bath of Huoxue Tongluo decoction(30 min, tid for 2wks,* n*=42)	No additional Tx. (n=37)	(1) Incidence rate (NCI-CTC; sensory neuropathy) (2)QoL (KPS) (3)Adverse events
Li, Cai [19]	2017	65 (0)	I:53 C:56	colorectal cancer	XELOX (d1-14, 3wks/cycle for 2 cycles)	Hand and foot bath, fumigation of Wenjing Huoxue formula(15 min, qd for 6 wk,* n*=34)	No additional Tx. (n=31)	(1)Incidence rate (Levi‘s grade) (2)QoL (KPS) (3)Adverse events
Lou [20]	2014	102(1)	I:59.84±*9.30 *C:57.94±*10.55*	Various types of cancer	Oxaliplatin based CTx.	Hand and foot bath of Wenjing Tongluo formula(20 min, bid for 7 days,* n*=67)	placbo(20 min, bid for 7 days,* n*=34)	(1)Clinical improvement (NCI-CTC; sensory neuropathy,) (2)NRS (pain) (3)NCCN (pain) (4) Adverse events
Qin [21]	2012	68 (0)	I:57.2±*8.6 *C:59.5±*7.9*	Various types of cancer	(1)Oxaliplatin/paclitaxel based CTx. (2)AD pro injection (80ml qd) (3) Thymopentin(1 mg qd) (for 14 days)	Hand and foot bath of Network Vessel-freeing Formula(30 min, qd for 14 days,* n*=34)	Cobamamide for intramuscular injection(1mg qd for 14 days, n=34)	(1)Clinical effectiveness (Nimodipine trichotomy) (2)Incidence rate (researcher's own criteria) (3)Adverse events
Shen [22]	2015	60 (0)	I:59.67±*12.72 *C:56.57±*11.32*	Various types of cancer	(1)Oxaliplatin based CTx. (2)Mecobalamin for intramuscular injection (0.5 mg tiw) (3)AD pro injection (50ml qd) (4)Thymopentin for subcutaneous injection(1.6 mg biw) (for 14 days)	Hand and foot bath of modified Huangqi-Guizhi Wuwu decoction(30 min, qd for 14 days,* n*=30)	No additional Tx. (*n*=30)	(1)Clinical improvement (NCI-CTC; sensory neuropathy,) (2)Clinical symptom (GPCR-NCM) (3)Adverse events
Tang [23]	2014	128(23)	I:59.46±*11.51 *C:61.16±*9.47*	Gastric and colorectal cancer	Oxaliplatin based CTx (for 6-8 cycles)	Hand and foot bath of Yangxue Wenjing Tongluo decoction ( during CTx, 20 min, bid,* n*=54)	No additional Tx. (*n*=51)	(1)Incidence rate (WHO grade) (2)Clinical symptom (TCSS) (3) Adverse events
Yi, Li [24]	2017	100 (0)	Not mentioned	colorectal cancer	Oxaliplatin based CTx.(21 d/cycle for 6 cycles)	Hand and foot bath of Tongluo Zhibi decoction (during CTx, 30 min, qd for 6 cycles*, n*=50)	Warm water for Hand and foot bath (during CTx, 30 min, qd for 6 cycles*, n*=50)	(1)Incidence rate (Levi‘s grade) (2)QoL (KPS) (3) Adverse events
Zhang [25]	2015	64 (0)	Not mentioned	Gastric and colorectal cancer	FOLFOX 4	Hand and foot bath of Tongjing, Huoxue formula (during CTx, 1000ml, 30 min, qd for 7 d*, n*=32)	Warm water for Hand and foot bath (during CTx, 1000ml, 30 min, qd for 7 d*, n*=32)	(1)Clinical improvement (Levi‘s grade, NCI-CTC) (2)QoL (KPS)
Feng [26]	2014	120 (0)	I:53.18±*13.61 *C:51.95±*11.37*	Various types of cancer	(1)Cisplatin based CTx. (2)Glutathione (1500mg/m^2^,d1-7)	Herbal Compress (for 7 d, n=60)	No additional Tx. (n=60)	(1)Incidence rate (WHO grade) (2)Duration time (3)Herbal related adverse events
Xu, Zhang [27]	2018	67 (0)	I:57.89±*11.54 *C:54.71±*12.24*	Various types of cancer	Not mentioned.	(1)Xiaotan Tongluo Gel for external use(1 mL/cm2,14 d/cycle for 2 cycles,* n*=36) (2)Mecobalamin tablets (0.5 mg tid)	(1)Placebo Gel for external use(1 mL/cm2, 14 d/cycle for 2 cycles,* n*=31) (2)Mecobalamin tablets (0.5 mg tid)	(1)Clinical improvement (NCI-CTC; sensory neuropathy,) (2)Clinical symptom (GPCR-NCM) (3) NCV (1)SNCV: median nerve/fibular nerve (2)MNCV: median nerve/ fibular nerve

*Herbal medicine for intravenous infusion*								
Chen, Huang [28]	2018	58 (0)	I:42-78 C:43-78	Gastric and colorectal cancer	(1)FOLFOX4(2wks/cycle for 4 cycles) (2)Methycobal Injection for Intramuscular injection (0.5 mg qd during CTx)	Astragalus injection for intravenous infusion(30ml qd during CTx, n=29)	No additional Tx. (n=29)	(1) Incidence rate (WHO grade) (2) QoL (KPS) (3) NCV (1)SNCV: median nerve/peroneus communis nerve (2)MNCV: median nerve/ Peroneus communis nerve
Cheng [29]	2014	36 (0)	I:46 C:49	Various types of cancer	Oxaliplatin based CTx. (130mg/m^2^,d1,21d/cycle for 4 cycles)	Tanshinone IIA Sodium Sulfonate Injection for intravenous infusion (80mg qd for d1-3,n=18)	(1)Tropisetron (5mg/d) (2)dxm (5mg/d) During CTx (n=18)	(1)Incidence rate (Levi‘s grade) (2)SNCV/MNCV (3)SOD/MDA
Cui [30]	2009	40 (0)	I:60 C:63	Gastric and colorectal cancer	Oxaliplatin based CTx.(130mg/m^2^,d1, for 1 cycle)	Astragalus injection for intravenous infusion(30ml qd for d1-7, n=20)	No additional Tx. (n=20)	(1)Incidence rate (Levi‘s grade) (2)NGF

*Herbal medicine for oral dosage form*								
Li, Sun [31]	2017	56 (0)	I: 58.89±*8.75*C: 57.71±*9.31*	Various types of cancer	n.c.	Granulas of Chinese Medicine of modified Huangqi-Guizhi Wuwu and Shentong Zhuyu(10g, bid for 1month,* n*=28)	Placebo(10g, bid for 1 month,* n*=28)	(1)Clinical improvement (NCI-CTC, sensory neuropathy,) (2)NRS (numbness) (3)ECOG PS (EORTC QLQ-C30) (4) Adverse events
Liu, Zhou [32]	2011	90 (5)	I:61.47±*9.05 *C:60.43±*9.48*	Various types of cancer	Oxaliplatin based CTx.(130mg/m^2^,d1, every 21 days/cycle, for 2 cycles)	Wangqi Guizhi Wuwu decoction(100ml, bid for 42 days, during CTx,* n*=28)	Mecobalamin (0.5 mg tid for 42 days,* n*=29)	(1) Clinical improvement (Levi‘s grade) (2)NCV (1)SNCV: median nerve/fibular nerve (2)MNCV: median nerve/ fibular nerve (3)Effective rate
Peng [33]	2015	96 (0)	I:57.2 C:55.7	Gastric and colorectal cancer	(1)mFOLFOX6(2 wks/cycle for 4 cycles) (2)Calcium gluconate, magnesium sulfate for injection (before CTx, for 2 days)	Danggui Sini Decoction combined with Yanghe Decoction(150ml, ( bid for 8 wks, during CTx,* n*=48)	No additional Tx. (n=48)	(1)Incidence rate (Levi‘s grade) (2) NCV (1)SNCV: median nerve/fibular nerve (2)MNCV: median nerve/ fibular nerve (3)Hemodynamic parameters
Chen, Yi [34]	2016	70 (0)	I:63.9±*7.9 *C:64.0±*8.1*	colorectal cancer	Oxaliplatin based CTx.(21 d/cycle for 4 cycles)	Tongluo Zhibi decoction(1wk before CTx- the end, n=35)	No additional Tx. (n=35)	(1)Incidence rate (researcher's own criteria) (2)NGF (3)SNCV/SNAPA
Shi [35]	2010	56 (0)	I:62.8 C:62.1	Gastric and colorectal cancer	FOLFOX 4 (for 4 cycles)	Wenjing Decoction (bid for 2 months, during CTx,* n*=30)	No additional Tx. (*n*=26)	Incidence rate (Levi‘s grade)
Xu [36]	2016	76 (0)	I:45.3±*5.8 *C:44.9±*5.5*	Ovarian cancer	TP(21 d/cycle for 6 cycles)	Modified Wangqi Guizhi Wuwu decoction(150ml, qd for 2 wks, during CTx,* n*=38)	Mecobalamin tablets (0.5 mg tid for 2 wks,* n*=38)	(1)Incidence rate (NCI-CTC; sensory neuropathy,) (2) NCV (1)SNCV: median nerve/fibular nerve (2)MNCV: median nerve/ fibular nerve
Yu, Su [37]	2014	50(1)	I:58.4±*5.3 *C:57.6±*4.7*	Various types of cancer	(1)TP(21 d/cycle for 6 cycles) (2)5-HT3 inhibitor	Jiawei Wangqi Guizhi Wuwu decoction (qd for 14 d/cycle, during CTx, for 6 cycles* n*=25)	Mecobalamin tablets (0.5 mg, tid for 14 d/cycle during CTx, for 6 cycles,* n*=24)	(1)Incidence rate (WHO grade) (2) NCV (1)SNCV: median nerve/fibular nerve (2)MNCV: median nerve/ fibular nerve

FOLFOX: folinic acid, fluorouracil, and oxaliplatin, XELOX: capecitabine and oxaliplatin, TP: paclitaxel and cisplatin, n.c.: no common treatment, Tx.: treatment, CTx.: chemotherapy, d:day, min:minute, qd: once daily, bid: twice a day, tid: three times a day, biw: twice a week; tiw: three times a week,

GPCR-NCM: Guiding Principles for Clinical Research of New Chinese Medicines, TCSS:Toronto Clinical Scoring System, NCI-CTC: National Cancer Institute Common Toxicity Criteria for Adverse Events,

EORTC QLQ-C30: European Organization for Research and Treatment of Cancer Quality of Life Questionnaire-30,

ECOG PS: Eastern Cooperative Oncology Group performance status,

NCCN: National Comprehensive Cancer Network Guidelines for Adult Cancer, WHO: world health organization,

QoL: quality of life, KPS: Karnofsky Performance Scale, PNCV: peripheral nerve conduction velocity, NCV: nerve conduction velocity, MNCV: motor nerve conduction velocity, SNCV: sensory nerve conduction velocity, SNAPA: Sensory nerve action potential amplitude, NGF: nerve growth factor, SOD: superoxide dismutase, MDA: malondialdehyde, NRS: Numerical Rating Scale.

**Table 2:** The characteristics of the formulas.

First author	Year	Name of the formula	Composition(daily dosage)	Jadad score
Chen, Mou	2015	Huoxue Tongluo decoction	Cassia Twig (10), Astragalus(25), Salvia(10), Peach Kernel(10), Safflower(5), Angelica Sinensis(10), Rhizoma ligustici chuanxiong(10), Millettia Reticulata(5), White Peony Root(30), Herba Lycopodti(30), Clematis Root(30)	4
Chen, Yi	2016	Tongluo Zhibi decoction	Angelica Sinensis(20),Rhizoma ligustici chuanxiong(15), Notopterygium(20), Trogopteroum feces(20), Prepared Monkshood(5), Radix Aconiti Kusnezoffii Preparata(5), Achyranthes Bidentata(20), Lanceolata(20), Loranthus Parasiticus(15), Glycyrrhiza(3)	4
Chen,Huang	2018	Astragalus injection		4
Cheng	2014		Tanshinone IIA	4
Cui	2009	Astragalus injection		4
Feng	2014	Herbal Compress	Mountain Spicy Tree Root and Rhizome(60), Millettia Reticulata(40), Aralia(180), Altingia chingii(60), large diamond(40), Caulis Tinosporae Sinensis(60), Bauhinia Championii Benth(60)	4
Li, Cai	2017	Wenjing Huoxue formula	Caulis Tinosporae Sinensis(30), Cassia Twig(30), Chinese Angelica Root(15), Chinese Mugwort Leaf(30), Mint(15), Biota Orientalis(30), Lu Lu Tong(30), Rhizoma ligustici chuanxiong(10)	4
Li, Sun	2017	Granulas of Chinese Medicine of modified Huangqi-Guizhi Wuwu and Shentong Zhuyu	Astragalus, Cassia Twig, White Peony Root, Ginger, Safflower, etc. (dosage not available)	6
Liu, Zhou	2011	Wangqi Guizhi Wuwu decoction	Astragalus(30), Cassia Twig (12), White Peony Root(15), Ginger(12), Jujube(15)	5
Lou	2014	Wenjing tongluo formula:	Geranium Herba, Aconiti Tuber, Cinnamomi Ramulus, Carthami Flos (the proportions are 4: 2: 3: 2)	7
Peng	2015	Danggui Sini Decoction combined with Yanghe Decoction	Cassia Twig(15), White Peony Root(15), Glycyrrhiza(3), Ginger(15), Jujube(15), Angelica Sinensis(10), Asarum(10), Dried Ginger(10), Antler Glue(10), Cinnamon(10), Seeds of Brassica Alba(10), Ephedra(10), Prepared Radix Rehmanniae(10)	4
Qin	2012	Network Vessel-freeing Formula	Cassia Twig(12), Astragalus(20), Salvia(15), Peach Kernel(12), Safflower(10), Angelica Sinensis(12), Rhizoma ligustici chuanxiong(15), Millettia Reticulata(30), White Peony Root(12), Zedoary Turmeric(10)	4
Shen	2015	Modified Huangqi-Guizhi Wuwu Decoction	Astragalus(50), White Peony Root(15), Cassia Twig(12), Dried Ginger(10), Jujube(10), Angelica Sinensis(12), Safflower(10), Millettia Reticulata(30), Clematis Root(10)	4
Shi	2010	Wenjing Decoction	Evodia Rutaecarpa(6-10), Angelica Sinensis(12), Rhizoma ligustici chuanxiong(10), White Peony Root(10), Lanceolata(30), Cassia Twig(6), Donkey Hide Gelatin(15), Ginger(2 slices), Cortex Moutan(10), Glycyrrhiza(6), Pinellia Ternate(10), Tuber of Dwarf Lilyturf(10), Astragalus(30)	4
Tang	2014	Yangxue Wenjing Tongluo Decoction	Astragalus(30), Angelica Sinensis(10), Aconite(10), Millettia Reticulata(30), Chinese mugwort leaf(10), Lu Lu Tong(10), Safflower(10)	4
Xu, Zhang	2018	Xiaotan Tongluo Gel	Arisaema Consanguineum, Pinelliae Tuber, Scorpion, Pleione Rhizome, Clematis Root, Cassia Twig, Safflower, Baked Licorice (the proportions are 5:5:2:5:5:5:3:2)	5
Xu	2016	Modified Wangqi Guizhi Wuwu Decoction	White Peony Root(12), Cassia Twig(12), Astragalus(25), Jujube(6), Ginger(15)	4
Yi, Li	2017	Tongluo Zhibi Decoction	Cassia Twig(15), Salvia(30), Red Peony Root(20), Rhizoma ligustici chuanxiong(20), Cynanchum Paniculatum(30), Clematis Root(30), Borneol(10), Asarum(10), North Papaya(20)	4
Yu, Su	2014	Jiawei Wangqi Guizhi Wuwu decoction	Astragalus(30), Cassia Twig(15), White Peony Root(15), Ginger(15), Jujube(25)	4
Zhang	2015	Tongjing, Huoxue formula	Epimedium(30), Geranium wilfordii Maxim(30), Cassia Twig(18), Rhizoma ligustici chuanxiong(18)	4

**Table 3:** The high frequency Chinese herbs.

English name	Chinese name	Counts	Frequency(%)
Cassia twig	Guizhi	14	10.53%
Astragalus	Huangqi	9	6.77%
White Peony Root	Baishao	9	6.77%
Angelica Sinensis	Danggui	7	5.26%
Rhizoma ligustici chuanxiong	Chuanxiong	7	5.26%
Safflower	Honghua	7	5.26%
Ginger	Shengjiang	6	4.51%
Millettia Reticulata	Jixueteng	5	3.76%
Jujube	Dazao	5	3.76%
Clematis root	Weilingxian	4	3%
Glycyrrhiza	Gancao	4	3%
Salvia	Danshen	3	2.26%
